# Cerebral microvascular and microstructural integrity is regionally altered in patients with systemic lupus erythematosus

**DOI:** 10.1186/s13075-020-02227-7

**Published:** 2020-06-08

**Authors:** Mark W. DiFrancesco, Gregory Lee, Mekibib Altaye, Dean W. Beebe, Jamie Meyers-Eaton, Hermine I. Brunner

**Affiliations:** 1 Imaging Research Center, Department of Radiology, Cincinnati Children’s Hospital Medical Center, University of Cincinnati College of Medicine, Cincinnati, OH 45229 USA; 2 Division of Biostatistics and Epidemiology, Cincinnati Children’s Hospital Medical Center, University of Cincinnati College of Medicine, Cincinnati, OH 45229 USA; 3 Division of Behavioral Medicine and Clinical Psychology, Department of Pediatrics, Cincinnati Children’s Hospital Medical Center, University of Cincinnati College of Medicine, Cincinnati, OH 45229 USA; 4 Division of Rheumatology, Department of Pediatrics, Cincinnati Children’s Hospital Medical Center, University of Cincinnati College of Medicine, Cincinnati, OH 45229 USA

**Keywords:** Systemic lupus erythematosus, Neurocognitive dysfunction, Neuroimaging, Magnetic resonance imaging, Diffusion-weighted imaging, Intravoxel incoherent motion

## Abstract

**Background:**

To measure regional brain microvascular and microstructural changes in childhood-onset SLE (cSLE) using diffusion-weighted imaging (DWI) at multiple *b* values and investigate relationships of those measures with neurocognitive function and disease activity.

**Methods:**

In this cross-sectional, case-control study, vascular volume fraction, effective diffusion, parenchymal diffusion, and blood flow parameters were regionally compared in cSLE patients and matched healthy controls. These markers of microvascular and microstructural integrity were derived by diffusion-weighted brain MRI and intravoxel incoherent motion (IVIM) modeling. Formal neurocognitive testing was completed focused on the domains of attention, visuoconstructional ability, working memory, and psychomotor speed. Test scores and measures of disease severity were regressed against regional microvascular integrity parameters among cSLE patients.

**Results:**

Formal cognitive testing confirmed normal cognitive ability among all cSLE patients included in the analysis (*n* = 11). Nevertheless, reduction in blood volume fraction coincided with increased effective diffusion and flow parameters in cSLE patients vs. controls in posterior brain regions including the cuneus and precuneus. Regional microvascular measures correlated (|*r*| = 0.54–0.66) with neuropsychiatric scores and disease activity among cSLE patients.

**Conclusions:**

There is imaging evidence, using IVIM, of degraded microvascular integrity in cSLE patients with normal cognitive ability. The observed regional changes correspond with posterior vascular border zones. These outcomes appear consistent with regional gray matter volume loss previously observed in cSLE patients with overt neurocognitive deficits, supporting the notion that adverse vascular changes precede loss of cognitive ability in cSLE. Longitudinal studies are needed to confirm the findings of this initial study.

## Background

Childhood-onset systemic lupus erythematosus (cSLE) is a systemic autoimmune disease often associated with neurocognitive deficits [1, 2]. Pathophysiological mechanisms of neurodegeneration by SLE are not well understood. Acquiring mechanistic insights could enable earlier and more accurate diagnosis, improved monitoring, and greater treatment efficacy. A plausible route of brain injury by SLE is through disruption of the neurovascular unit (NVU), a physiological construct describing the interplay between neurons, glia, and supporting vasculature. Anti-endothelial antibodies can drive immune responses in SLE that degrade vascular structural and functional integrity [3, 4]. A key component of the NVU is the blood-brain barrier (BBB), the selectively permeable interface between blood and parenchymal cells. Recently, we and others have reported regionally increased permeability of the BBB in SLE in comparison to matched healthy controls [5, 6]. BBB disruption can allow brain-reactive proteins to enter the parenchyma and contribute to neuronal pathology [7, 8]. Various studies have also detected alteration of cerebral blood perfusion in association with SLE [9, 10].

This study continues assessment of NVU degradation associated with SLE by applying intravoxel incoherent motion (IVIM) analysis to diffusion-weighted imaging (DWI) data at multiple diffusion weightings to determine alterations of vascular topology and flow. We compare regional microstructural and microvascular parameters, derived from the IVIM model, in cSLE patients to those of matched healthy controls, and relate the degree of parametric alteration to clinical and cognitive characteristics among members of the cSLE group. We hypothesized that there is regional loss of vascular fine structure and altered flow in cSLE compared to controls, hence a relative decrease in vascular integrity, which correlates with neurocognitive function and disease activity.

## Methods

### Subjects

This report focuses on analysis of DWI data ancillary to a previously reported study of regional BBB differences between SLE patients and matched healthy controls [6]. The study recruited 12 patients diagnosed with SLE [11] prior to age 18 years (cSLE) from a tertiary pediatric rheumatology clinic, with apparently normal cognitive function (see Additional File 1). Exclusion criteria were structural brain abnormalities, neuropathies, movement disorders or seizures, and medications (besides oral corticosteroids) that could influence cognition. One healthy control subject was enrolled for each cSLE patient, matched by age (within 1 year), gender, ethnicity, and socioeconomic status. The study secured Institutional Review Board approval, with full informed consent obtained from all participants.

### Subject assessments

Demographic data including age, gender, ethnicity, and annual family income were collected. ZIP code-based median family incomes were compiled using the US Census Bureau report, 2009–2013 (http://factfinder.census.gov). Clinical characteristics including body mass index (BMI) and blood pressure served as indices of metabolic and cardiovascular status. Disease activity and damage were characterized in cSLE patients by the SLE disease activity index (SLEDAI) and Systemic Lupus International Collaborating Clinics/ACR Damage Index (SDI), respectively.

Four cognitive domains often impacted by cSLE, i.e., attention, working memory, visuoconstructional ability (VCA), and psychomotor speed (PMS) [12, 13], were assessed by formal neurocognitive testing [14] in all participants. Details are provided in Additional File 2. Performance in each cognitive domain was expressed as a *Z*-score. We designated a participant as having clinically relevant neurocognitive deficits if at least two domain *Z*-scores were below − 1 or at least one domain *Z*-score was below − 2 [15].

### Imaging acquisition and image processing

The complete imaging session included four types of sequences: (1) a high-resolution T1-weighted structural image as anatomic reference for all parametric maps and as basis for tissue segmentation, (2) localized blood dynamics via arterial spin labeling (ASL) at 15 label/post-label delay durations, (3) DWI in 3 orthogonal directions at 15 *b* values for regional estimation of microvascular and microstructural parameters, and (4) tissue T1 quantification by inversion recovery (IR) acquisitions at 10 inversion times (TI). Imaging took place on a 3-T Philips Achieva scanner (Philips Research, Eindhoven, Netherlands) equipped with a 32-channel head coil. The complete imaging session had a duration of approximately 70 min, including imaging pauses between successive individual sequences. Results from the DWI series are the focus of this report.

#### Imaging parameters

The structural image was obtained from a high-resolution, T1-weighted, inversion-prepared 3-dimensional whole brain scan for each subject [16]. Parameters for this scan were as follows: repetition time (TR) = 6.8 ms, echo time (TE) = 2.9 ms, inversion recovery time (TI) = 904 ms, and field of view (FOV) = 176 × 256 × 256 mm, with 1 mm isotropic resolution (total time 6 min, 46 s). DWI data were acquired axially with the geometric parameters: FOV = 240 × 240 × 120 mm, with resolution 3.75 × 3.75 × 5 mm. DWI data were acquired with diffusion gradients applied in three orthogonal directions with echo-planar imaging (EPI) readout and cerebrospinal fluid (CSF) suppression via initial inversion pulse (TR/TE/TI = 3536/48/2200 ms, number of averages (NSA) = 2, duration = 13 min, 5 s). The diffusion gradients were applied at 15 different *b* values (0, 5, 10, 20, 30, 40, 60, 80, 120, 160, 200, 300, 500, 750, and 1000 s/mm^2^).

#### Image pre-processing

DWI pre-processing began with the average of the 3 gradient directions for each *b* value. These 15 images underwent eddy-current correction and alignment to the *b* = 0 image under the FDT (FMRIB’s Diffusion Toolbox) in FSL (FMRIB Software Library), an analytical tools library [17]. Using SPM12 software [18] under the Matlab environment (The Mathworks, Natick, MA, USA), the T1-weighted structural image was co-registered to the *b* = 0 image before normalization to the MNI (Montreal Neurological Institute) standard space and tissue segmentation were achieved in a combined process based on gray matter, white matter, and cerebrospinal fluid priors [19]. The resulting nonlinear normalization transformation was applied to the DWI series with preservation of the original voxel size. Finally, the DWI data were smoothed with an 8-mm Gaussian kernel in SPM12.

Microvascular and microstructural properties were assessed per voxel by fitting the DWI signal vs. *b* value to a bi-exponential diffusion model using in-house Matlab scripts. The intravoxel incoherent motion model (IVIM) was applied for fractional change in signal, *S*, as a function of *b* value [20, 21]:

$$ \frac{dS(b)}{S}=\left(1-{v}_{bw}\right)\exp \left(- bD\right)+{v}_{bw}\exp \left(-b\left(D+{D}^{\ast}\right)\right), $$

where *D* is the diffusion coefficient of water in bulk tissue, *D*^∗^ is the effective diffusion coefficient of water in randomly-oriented microvasculature, and *v*_*bw*_ is the fraction of water molecules in blood. Another parameter of interest, derived from the IVIM parameters, is the product *D*^∗^ × *v*_*bw*_, which indicates blood flow [20, 21].

### Statistical analysis

Descriptive statistics were used for comparison of demographic and clinical information between the two groups (cSLE vs. controls), using the independent sample *t* test or chi-square test as appropriate in SAS® software version 9.4 (SAS Institute Inc., Cary, NC, USA). Voxel-wise comparisons of *v*_*bw*_, *D*, *D*^∗^, and the flow-related product, *D*^∗^ × *v*_*bw*_, were made between the cSLE and control groups via independent sample *t* test in SPM12 [18]. A nominal *T*-score threshold was applied per voxel, resulting in contiguous voxel clusters that were tested for significance at *p* < 0.05, family-wise error corrected for multiple comparisons based on random field theory [22].

Correlations between each IVIM parameter and neurocognitive summary domain *Z*-scores and disease activity (SLEDAI score) were explored among the cSLE patients, restricted to brain regions in which the IVIM parameters were found to be impacted by cSLE. In consideration of the pilot nature of this study and corresponding small sample size, we report statistical significance at the *p* < 0.05 level without correction for performing multiple tests across different IVIM parameters and different cognitive or disease activity scores.

## Results

### Participant characteristics

One control subject and one cSLE subject were excluded from analysis due to image artifact on DWI, likely due to motion. As expected based on the matching strategy, demographic characteristics and cognitive performance of the remaining 11 cSLE patients and 11 control subjects were similar (Table 1). Among these participants, none were smokers, with the exception of one of the controls. None of the cSLE patients met criteria for clinically relevant neurocognitive deficits or carried a diagnosis of neuropsychiatric involvement with cSLE.

**Table 1:** Baseline information for SLE patients and matched controls

Variable	SLE patients (*n* = 11)	Matched controls (*n* = 11)	*p* value^†^
**Demographics**			
Age, years*	19.5 ± 4.2	17.0 ± 6.8	0.312
Females (*n*)	10	10	1.000
Race (African American, *n*)^@^	6	6	1.000
Income (*n* < $50,000/year)**	6	8	0.658
Median family income ($)* (US census based on ZIP)	64,090.91 ± 41,690.42	59,857.09 ± 9700.90	0.746
**Physical exam findings**			
Body mass index (BMI)*	23.0 ± 4.9	25.9 ± 4.1	0.148
Blood pressure (mmHg)			
Systolic*	115.4 ± 15.4	120.6 ± 10.9	0.372
Diastolic*	72.2 ± 7.3	69.4 ± 5.3	0.316
**Neurocognitive assessment**^**#**^			
Domains (*Z*-score)*			
(1)Working memory	0.06 ± 0.72	− 0.34 ± 0.65	0.187
(2)Psychomotor speed	0.15 ± 0.88	− 0.15 ± 0.55	0.349
(3)Attention	0.34 ± 0.77	− 0.07 ± 0.90	0.264
(4)Visuoconstructional ability	0.46 ± 0.45	− 0.15 ± 0.56	0.610
**SLE measures**^‡^			
Disease duration, years*	6.3 ± 4.3	–	
ds DNA positive (*n*)	5	–	
Complement C3* (mg/dl)	96.9 ± 29.3	–	
Complement C4* (mg/dl)	17.4 ± 8.9	–	
SLEDAI*	5.7 ± 4.7	–	
SLICC-ACR damage index (median (range))	0 (0–4)	–	
**Prednisone use (*n*)**			
None	2	–	
≤ 5 mg/day	7	–	
> 5 mg/day	2	–	

The use of *n* indicates the number of patients identified in that particular group or subgroup

*Expressed in mean ± standard deviation (SD) values

**Income differences calculated using independent sample *t* test comparing mean incomes between the two groups

^†^*p* values are based on independent sample *t* tests comparing the two groups

^‡^Here, SLEDAI (SLE Disease Activity Index); SLICC-ACR (Systemic Lupus International Collaborating Clinics/American College of Rheumatology) Damage Index is a measure expressed in their calculated value

^@^Racial distribution for the non-African Americans included Caucasian (3) and Asian (2) for the c-SLE group and Caucasian (5) for the healthy controls

^#^Cognitive assessment used the following tests: (1) working memory [Wechsler Intelligence Scale for Children (WISC IV) Digit Span and Letter-Number Sequencing], (2) psychomotor speed [WISC IV Coding and Symbol Search; Conners’ Continuous Performance Test (CPT) II reaction time parameter], (3) attention [Conners’ CPT II Stroop Color and Word Test], and (4) visuoconstructional ability [Wechsler Abbreviated Scale of Intelligence Block Design Test, Kaufman Assessment Battery for Children, Second Edition Block Counting, Gestalt Closure]

All cSLE patients were taking hydroxychloroquine, and most were also taking low-dose prednisone [*n* = 9, mean (SD) = 5.8 (2.4) mg/day]. A variety of immunosuppressive medications were in use by the cSLE patients (3 mycophenolate mofetil, 2 leflunomide, 1 tocilizumab, 1 methotrexate).

### Group comparison and correlation outcomes

**Fig. 1:**
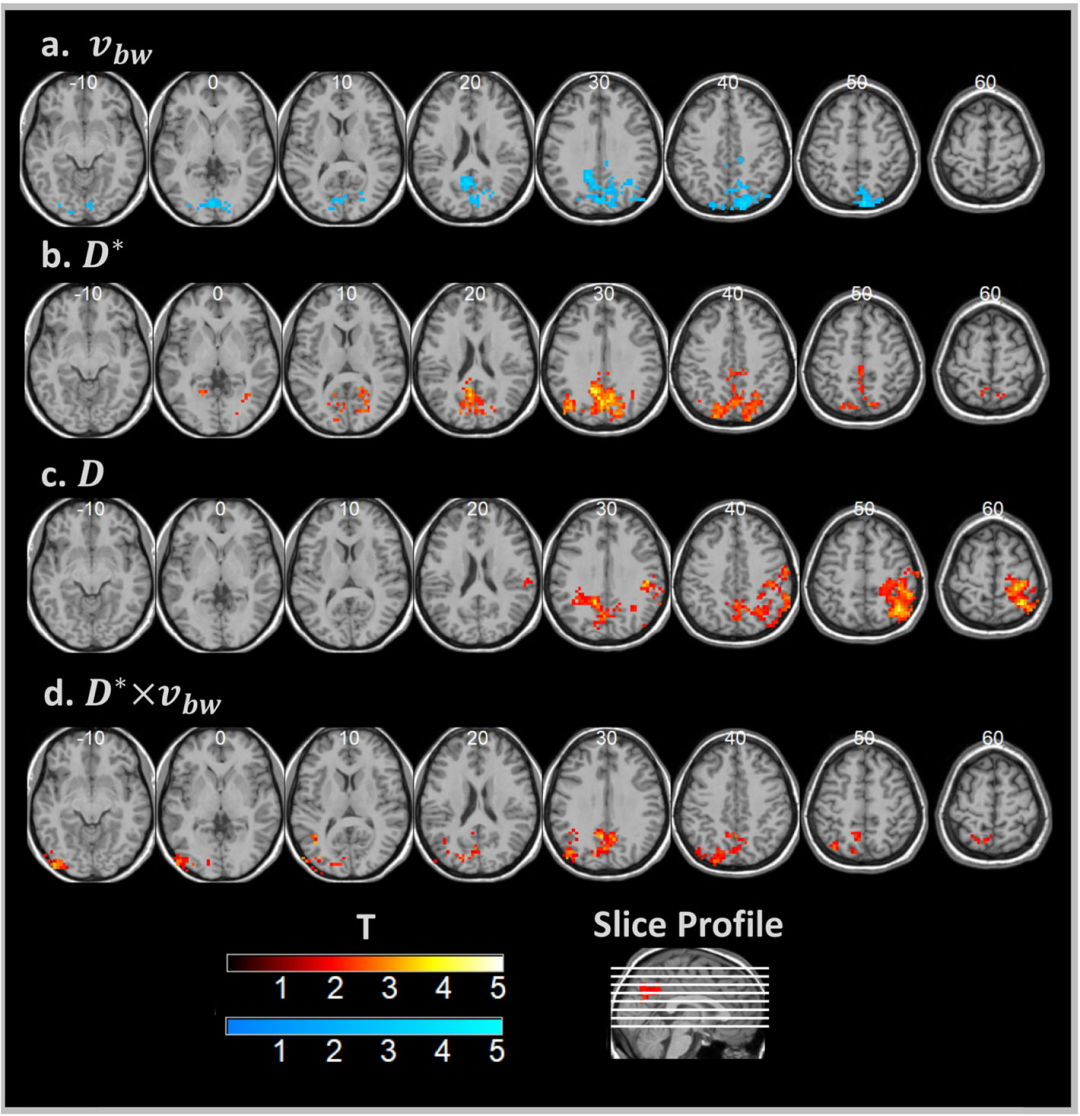
Group difference *T*-score maps. Hot colors indicate cSLE group > healthy control group, and cool colors indicate healthy control group > cSLE group. All clusters significant at the cluster-level *p* < 0.05, family-wise error corrected. **a** Blood volume fraction. **b** Effective diffusion coefficient. **c** Parenchymal diffusion coefficient. **d** Flow-dependent effective diffusion × blood volume fraction product. MNI *z* coordinate of each slice is indicated. Neurological orientation convention is used

Group comparisons of IVIM parameter maps, shown in Fig. 1, revealed clusters of voxels with significantly greater *D*^∗^ and *D*^∗^ × *v*_*bw*_ for cSLE patients compared to controls in the cuneus, precuneus, superior occipital, and posterior cingulate regions. Tissue diffusion, *D*, was also greater in the cSLE group for the precuneus and right inferior parietal areas. Conversely, controls were found to have higher values of *v*_*bw*_ compared to cSLE patients in the cuneus, precuneus, calcarine, and mid-occipital regions. A single posterior cluster was formed in each comparison, reaching cluster-level significance at corrected *p* < 0.05 after voxel-wise thresholding at *T*-scores ranging from 1.7 to 2.0, as indicated in Fig. 1 and detailed in Table 2.

**Table 2:** Voxel cluster characteristics (cluster-level *p* < 0.05 family-wise error corrected)

IVIM parameter	Contrast	Peak location [*x*, *y*, *z*] mm	T-score threshold*	Cluster size [voxels]	Anatomic regions
*v*_*bw*_	SLE < HC	[8, − 82, 50]	2.0	476	Right sup. parietal, mid. occipital, bilateral sup. occipital, precuneus, cuneus, calcarine, lingual, mid. cingulate
*D*^∗^	SLE > HC	[− 7, − 56, 25]	2.0	629	Bilateral sup. parietal, mid. occipital, sup. occipital, angular, precuneus, cuneus, mid. cingulate, post. cingulate, calcarine, lingual
*D*	SLE > HC	[38, − 52, 55]	1.8	731	Right postcentral, sup. parietal, inf. parietal, angular, supramarginal, bilateral precuneus, cuneus
*D*^∗^ × *v*_*bw*_	SLE > HC	[− 52, − 86, − 5]	1.7	365	Left sup. parietal, inf. occipital, mid. occipital, sup. occipital, angular, lingual, bilateral precuneus, post. cingulate, calcarine
Intersection	–	–	–	29	Left cuneus, post. cingulate, bilateral precuneus

*v*_*bw*_ = blood volume fraction, *D*^∗^ = effective diffusion coefficient, *D* = parenchymal diffusion coefficient, *D*^∗^ × *v*_*bw*_ = flow parameter, Intersection = region common to all IVIM parameter clusters

*Nominal voxel threshold used to generate significant cluster

**Fig. 2:**
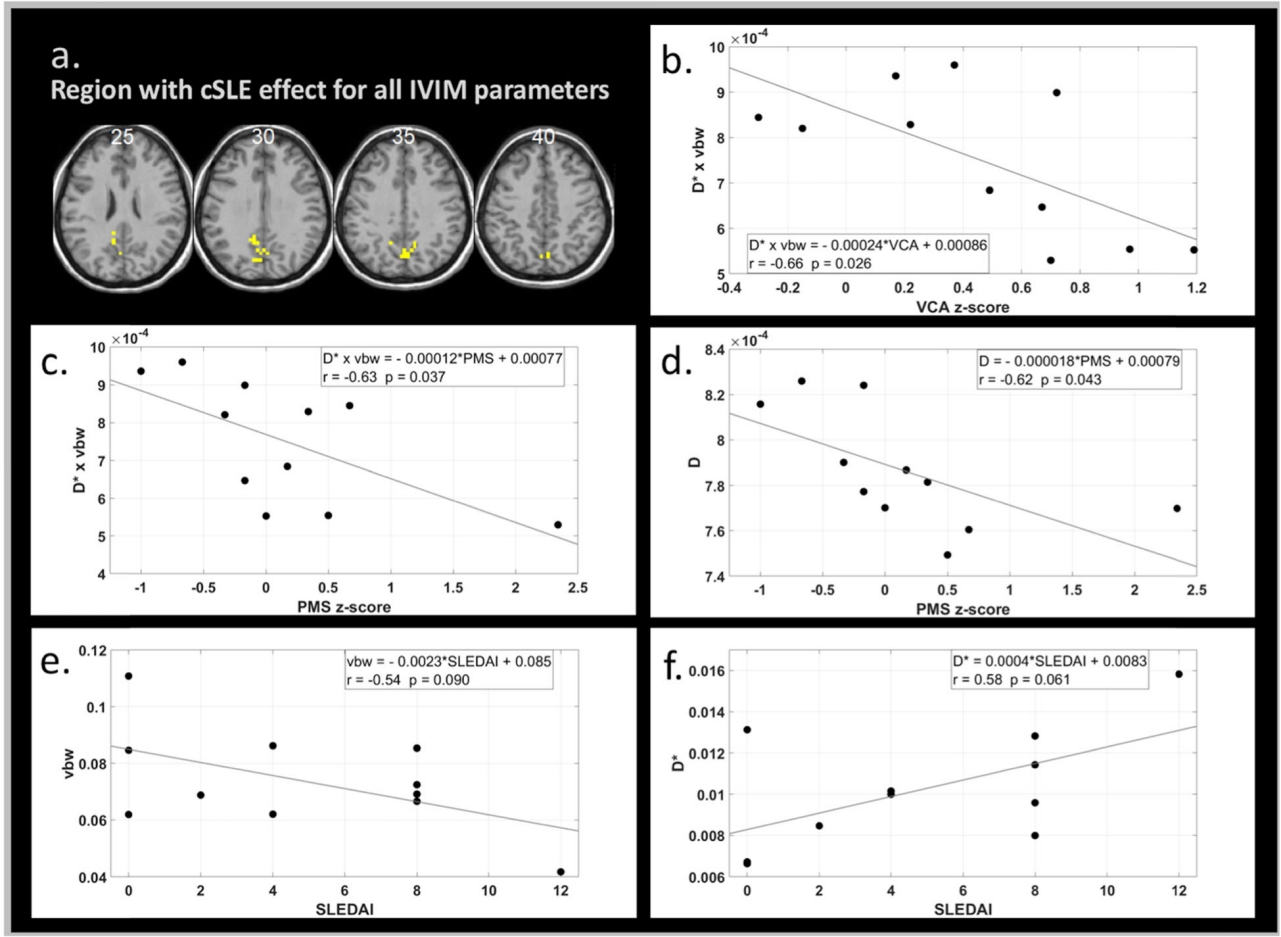
Correlation analyses of median IVIM parameter value in a brain region formed by the intersection of IVIM parameter change clusters for the cSLE group vs. healthy control group. **a** The intersection region shown in yellow. **b** Median flow parameter vs. VCA *z*-score. **c** Median flow parameter vs. PMS *z*-score. **d** Median parenchymal diffusion coefficient vs. PMS *z*-score. **e** Median blood volume fraction vs. SLEDAI score. **f** Median effective diffusion coefficient vs. SLEDAI score. MNI *z* coordinate of each slice in **a** is indicated, and neurological convention is used. For each plot **b**–**f**, the regression equation, *r* value, and *p* value are provided

Intersection of the significant clusters for cSLE vs. controls for all four IVIM parameters resulted in a midline posterior region in the precuneus. This region, depicted in Fig. 2a, exhibits all of the microstructural and microvascular changes with cSLE outlined above.

### Associations of imaging parameters with cSLE features and cognitive performance

Focusing on the intersection region, shown in Fig. 2a, where the spectrum of IVIM parameters was commonly impacted by cSLE, we found some IVIM parameters to be correlated with cognitive performance or with SLEDAI scores. Specifically, the median flow-related product, *D*^∗^ × *v*_*bw*_, within the intersection region was inversely correlated with VCA domain *Z*-scores (Fig. 2b). The median flow-related product and diffusion, *D*, each had a significant inverse linear relationship with PMS *Z*-scores (Fig. 2c, d d). Trending significance (*p* < 0.1) was observed in the correlations of *v*_*bw*_ and *D*^∗^ with SLEDAI scores, with *v*_*bw*_ decreasing and *D*^∗^ increasing with increasing SLEDAI scores.

## Discussion

We present imaging evidence of altered tissue and vascular microstructure in cSLE using IVIM analysis. Parietal regions of the brain were found to have augmented apparent diffusion, *D*^∗^, and flow-related product, *D*^∗^ × *v*_*bw*_, coupled with decreased blood volume fraction, *v*_*bw*_, among the cSLE patients. This combination of vascular parametric alterations suggests regional loss of vascular fine structure or ramification resulting in net reduction of cerebral blood volume, but increased rate of blood flow. The increased rate of blood flow may set the stage for vasogenic edema as is reflected by the observed increase in the parenchymal diffusion parameter, *D*. Notably, the posterior regions with these parametric changes were previously found to develop gray matter loss in SLE patients with overt clinically relevant neurocognitive deficits [15]. In line with our previous findings of associations of increased BBB permeability with cognitive performance in cSLE patients [6], the microvascular abnormalities reported here seem to precede the onset of clinically overt clinically relevant neurocognitive deficits.

Investigations of cerebral blood flow using MRI methods such as arterial spin labeling [9] and dynamic susceptibility contrast [23], or radioactive tracer methods such as single photon emission computed tomography (SPECT) [24] have shown inconsistent results for SLE. While some studies have reported cerebral hypoperfusion with SLE [9, 24, 25], others reported that SLE patients have increased regional cerebral blood flow compared to controls [10, 23, 26, 27]. The cSLE-associated increase in the blood flow-related product *D*^∗^ × *v*_*bw*_ found in posterior brain regions in this study supports hyperperfusion, at least in the absence of clinically relevant neurocognitive deficits.

The general pattern of differences in IVIM parameters between the cSLE and healthy control groups uncovered in this study corresponds to the confluence of border zones between the anterior, middle, and posterior cerebral artery territories [28, 29]. As in ischemic stroke [30], these regions are particularly vulnerable to early microvascular degradation, given the preponderance of distal vascular branches. Imaging evidence from this study places the earliest microvascular changes, in cSLE patients prior to exhibiting neuropsychiatric symptoms, specifically in or near posterior border zones.

The posterior border zone distribution of parenchymal diffusion increases and microvascular changes associated with cSLE is also consistent with features of posterior reversible encephalopathy syndrome (PRES) [31], a condition thought to result from endothelial dysfunction and accompanied by vasogenic edema. Indeed, circumstances that can lead to PRES include SLE, hypertension, and use of certain immunosuppressant medication [32]. Thus, our results suggest that despite the lack of clinically relevant neurocognitive deficits, other neurological symptoms, or hypertension (Table 1), there is subtle dysfunction of brain blood flow autoregulation in the vulnerable posterior zones for our cohort of cSLE patients.

Correlations were observed between various IVIM measures and cognitive performance scores or disease activity among the cSLE patients. The increased mean effective diffusion, *D*^∗^, and decreased blood volume fraction, *v*_*bw*_, found in the cSLE group compared to controls were associated with greater disease activity (SLEDAI). The flow-related product and parenchymal diffusion increases in cSLE were associated with diminished performance in visuoconstructional ability and psychomotor speed, both of which rely on parietal function [12, 33, 34].

Our study should be viewed in light of certain limitations. We based our results on a small sample of cSLE patients, which constrained statistical power. Additionally, the impact of steroid use on the neurovascular structure and function in the cSLE group must be taken into consideration. We can point out, however, that most of the included cSLE patients were treated with low-dose steroids, and our previous study suggests that oral steroids have little effect on regional gray matter volume in cSLE [15]. Though we cannot exclude that steroid use influences the microstructural and microvascular integrity, their anti-inflammatory effects likely diminished rather than enhanced any differences of IVIM parameters in the cSLE group compared to healthy controls. Studies focused on the specific impact of corticosteroids on neurovasculature are needed to rule out or correct for potentially confounding changes on imaging parameters like those from the IVIM model.

The findings of this study also motivate further studies to explore association of IVIM parameters in SLE patients with biomarkers such as anti-NR2 [35, 36] and anti-ribosomal P antibody [37–39] levels, which are both traditionally thought to be related to neuropsychiatric SLE activity.

## Conclusions

In summary, to the best of our knowledge, we are the first to apply DWI and IVIM analyses to assess cerebral microvascular changes in cSLE. We have identified posterior brain regions with a combination of vascular and parenchymal alterations in cSLE patients with normal cognitive ability, which overlay areas previously associated with functional and structural alterations with cSLE-associated NCD. These findings support the notion that subtle loss of microvascular integrity, especially in watershed zones between vascular territories, precedes structural brain changes and deterioration of cognitive performance in cSLE. Longitudinal studies are required to explore whether these DWI-derived parameters can combine to serve as an imaging biomarker that predicts development of neuropsychiatric SLE.

## Supplementary information

**Additional file 1: Table S1.** Revised ACR Classification Criteria* for SLE. A summary of the number of patients meeting various ACR classification criteria.

**Additional file 2: Table S2.** Cognitive Assessment – Domains and Tests Used. Details of the various cognitive domains assessed for the study participants.

## Data Availability

The datasets used and/or analyzed during the current study are available from the corresponding author on reasonable request.

## Abbreviations

cSLE: Childhood-onset systemic lupus erythematosus
DWI: Diffusion-weighted imaging
IVIM: Intravoxel incoherent motion
NVU: Neurovascular unit
BBB: Blood-brain barrier
SLE: Systemic lupus erythematosus
BMI: Body mass index
SLEDAI: SLE disease activity index
SDI: Systemic Lupus International Collaborating Clinics/ACR Damage Index
VCA: Visuoconstructional ability
PMS: Psychomotor speed
ASL: Arterial spin labeling
IR: Inversion recovery
TI: Inversion time
TR: Repetition time
TE: Echo time
EPI: Echo-planar imaging
FOV: Field of view
NSA: Number of averages
CSF: Cerebrospinal fluid
FMRIB: Oxford Centre for Functional MRI of the Brain
FDT: FMRIB’s Diffusion Toolbox
FSL: FMRIB Software Library
SPECT: Single photon emission computed tomography
PRES: Posterior reversible

## References

[1] Sibbitt WL, Brandt JR, Johnson CR, Maldonado ME, Patel SR, Ford CC, Bankhurst AD, Brooks WM (2002). The incidence and prevalence of neuropsychiatric syndromes in pediatric onset systemic lupus erythematosus. J Rheumatol.

[2] Tucker LB, Menon S, Schaller JG, Isenberg DA (1995). Adult- and childhood-onset systemic lupus erythematosus: a comparison of onset, clinical features, serology, and outcome. Br J Rheumatol.

[3] Legendre P, Regent A, Thiebault M, Mouthon L (2017). Anti-endothelial cell antibodies in vasculitis: a systematic review. Autoimmun Rev.

[4] Scolding NJ, Joseph FG (2002). The neuropathology and pathogenesis of systemic lupus erythematosus. Neuropathol Appl Neurobiol.

[5] Chi JM, Mackay M, Hoang A, Cheng K, Aranow C, Ivanidze J, Volpe B, Diamond B, Sanelli PC (2019). Alterations in blood-brain barrier permeability in patients with systemic lupus erythematosus. AJNR Am J Neuroradiol.

[6] Gulati G, Jones JT, Lee G, Altaye M, Beebe DW, Meyers-Eaton J, Wiley K, Brunner HI, DiFrancesco MW (2017). Altered blood-brain barrier permeability in patients with systemic lupus erythematosus: a novel imaging approach. Arthritis Care Res (Hoboken).

[7] Abbott NJ, Mendonca LL, Dolman DE (2003). The blood-brain barrier in systemic lupus erythematosus. Lupus.

[8] Stock AD, Gelb S, Pasternak O, Ben-Zvi A, Putterman C (2017). The blood brain barrier and neuropsychiatric lupus: new perspectives in light of advances in understanding the neuroimmune interface. Autoimmun Rev.

[9] Jia J, Xie J, Li H, Wei H, Li X, Hu J, Meng D, Zhang Y, Zhang X (2019). Cerebral blood flow abnormalities in neuropsychiatric systemic lupus erythematosus. Lupus.

[10] Wang PI, Cagnoli PC, McCune WJ, Schmidt-Wilcke T, Lowe SE, Graft CC, Gebarski SS, Chenevert TL, Khalatbari S, Myles JD, Watcharotone K, Cronin P, Sundgren PC (2012). Perfusion-weighted MR imaging in cerebral lupus erythematosus. Acad Radiol.

[11] Hochberg MC (1997). Updating the American College of Rheumatology revised criteria for the classification of systemic lupus erythematosus. Arthritis Rheum.

[12] DiFrancesco MW, Gitelman DR, Klein-Gitelman MS, Sagcal-Gironella AC, Zelko F, Beebe D, Parrish T, Hummel J, Ying J, Brunner HI (2013). Functional neuronal network activity differs with cognitive dysfunction in childhood-onset systemic lupus erythematosus. Arthritis Res Ther.

[13] DiFrancesco MW, Holland SK, Ris MD, Adler CM, Nelson S, DelBello MP, Altaye M, Brunner HI (2007). Functional magnetic resonance imaging assessment of cognitive function in childhood-onset systemic lupus erythematosus: a pilot study. Arthritis Rheum.

[14] Ross GS, Zelko F, Klein-Gitelman M, Levy DM, Muscal E, Schanberg LE, Anthony K, Brunner HI (2010). A proposed framework to standardize the neurocognitive assessment of patients with pediatric systemic lupus erythematosus. Arthritis Care Res (Hoboken).

[15] Gitelman DR, Klein-Gitelman MS, Ying J, Sagcal-Gironella AC, Zelko F, Beebe DW, Difrancesco M, Parrish T, Hummel J, Beckwith T, Brunner HI (2013). Brain morphometric changes associated with childhood-onset systemic lupus erythematosus and neurocognitive deficit. Arthritis Rheum.

[16] Mugler JP, Brookeman JR (1990). Three-dimensional magnetization-prepared rapid gradient-echo imaging (3D MP RAGE). Magn Reson Med.

[17] FSL: FMRIB Analysis Group: https://fsl.fmrib.ox.ac.uk/fsl/fslwiki/FSL.

[18] SPM12: Wellcome Trust Centre for Neuroimaging: http://www.fil.ion.ucl.ac.uk/spm/.

[19] Ashburner J, Friston KJ (2005). Unified segmentation. Neuroimage.

[20] Le Bihan D, Breton E, Lallemand D, Aubin ML, Vignaud J, Laval-Jeantet M (1988). Separation of diffusion and perfusion in intravoxel incoherent motion MR imaging. Radiology.

[21] Le Bihan D, Breton E, Lallemand D, Grenier P, Cabanis E, Laval-Jeantet M (1986). MR imaging of intravoxel incoherent motions: application to diffusion and perfusion in neurologic disorders. Radiology.

[22] Nichols T, Hayasaka S (2003). Controlling the familywise error rate in functional neuroimaging: a comparative review. Stat Methods Med Res.

[23] Gasparovic CM, Roldan CA, Sibbitt WL, Qualls CR, Mullins PG, Sharrar JM, Yamamoto JJ, Bockholt HJ (2010). Elevated cerebral blood flow and volume in systemic lupus measured by dynamic susceptibility contrast magnetic resonance imaging. J Rheumatol.

[24] Huang WS, Chiu PY, Tsai CH, Kao A, Lee CC (2002). Objective evidence of abnormal regional cerebral blood flow in patients with systemic lupus erythematosus on Tc-99m ECD brain SPECT. Rheumatol Int.

[25] Oda K, Matsushima E, Okubo Y, Ohta K, Murata Y, Koike R, Miyasaka N, Kato M (2005). Abnormal regional cerebral blood flow in systemic lupus erythematosus patients with psychiatric symptoms. J Clin Psychiatry.

[26] Gasparovic C, Qualls C, Greene ER, Sibbitt WL, Roldan CA (2012). Blood pressure and vascular dysfunction underlie elevated cerebral blood flow in systemic lupus erythematosus. J Rheumatol.

[27] Greene ER, Yonan KA, Sharrar JM, Sibbitt WL, Roldan CA (2012). Middle cerebral artery resistivity and pulsatility indices in systemic lupus erythematosus: evidence for hyperperfusion. Lupus.

[28] Savoiardo M (1986). The vascular territories of the carotid and vertebrobasilar systems. Diagrams based on CT studies of infarcts. Ital J Neurol Sci.

[29] Tatu L, Moulin T, Bogousslavsky J, Duvernoy H (1998). Arterial territories of the human brain. Neurology.

[30] Kapasi A, Leurgans SE, James BD, Boyle PA, Arvanitakis Z, Nag S, Bennett DA, Buchman AS, Schneider JA (2018). Watershed microinfarct pathology and cognition in older persons. Neurobiol Aging.

[31] Bartynski WS, Boardman JF (2007). Distinct imaging patterns and lesion distribution in posterior reversible encephalopathy syndrome. AJNR Am J Neuroradiol.

[32] Hugonnet E, Da Ines D, Boby H, Claise B, Petitcolin V, Lannareix V, Garcier JM (2013). Posterior reversible encephalopathy syndrome (PRES): features on CT and MR imaging. Diagn Interv Imaging.

[33] Hwang M, Tudorascu DL, Nunley K, Karim H, Aizenstein HJ, Orchard TJ, Rosano C (2016). Brain activation and psychomotor speed in middle-aged patients with type 1 diabetes: relationships with hyperglycemia and brain small vessel disease. J Diabetes Research.

[34] Liberg B, Adler M, Jonsson T, Landen M, Rahm C, Wahlund LO, Wiberg-Kristoffersen M, Wahlund B (2013). Motor imagery in bipolar depression with slowed movement. J Nerv Ment Dis.

[35] Arinuma Y, Yanagida T, Hirohata S (2008). Association of cerebrospinal fluid anti-NR2 glutamate receptor antibodies with diffuse neuropsychiatric systemic lupus erythematosus. Arthritis Rheum.

[36] Wang JY, Zhao YH, Zhang JH, Lei HW (2019). Anti-N-methyl-D-aspartic acid receptor 2 (anti-NR2) antibody in neuropsychiatric lupus serum damages the blood-brain barrier and enters the brain. Med Sci Monit.

[37] Bravo-Zehnder M, Toledo EM, Segovia-Miranda F, Serrano FG, Benito MJ, Metz C, Retamal C, Alvarez A, Massardo L, Inestrosa NC, Gonzalez A (2015). Anti-ribosomal P protein autoantibodies from patients with neuropsychiatric lupus impair memory in mice. Arthritis Rheumatol.

[38] Gonzalez A, Massardo L (2018). Antibodies and the brain: antiribosomal P protein antibody and the clinical effects in patients with systemic lupus erythematosus. Curr Opin Neurol.

[39] Massardo L, Bravo-Zehnder M, Calderón J, Flores P, Padilla O, Aguirre JM, Scoriels L, González A (2015). Anti-N-methyl-D-aspartate receptor and anti-ribosomal-P autoantibodies contribute to cognitive dysfunction in systemic lupus erythematosus. Lupus.

