# Supplementary material for: Cerebral microvascular and microstructural integrity is regionally altered in patients with systemic lupus erythematosus
Source: Arthritis Res Ther. 2020 Jun 8;22:135. doi: 10.1186/s13075-020-02227-7 (PMC7281933; doi:10.1186/s13075-020-02227-7)
Supplement: Supplementary file 1 — Additional file 1: Table S1. Revised ACR Classification Criteria* for SLE. A summary of the number of patients meeting various ACR classification criteria. [file 13075_2020_2227_MOESM1_ESM.docx]

SUPPLEMENTARY TABLE 1. Revised ACR Classification Criteria* for SLE

(Total cSLE patients = 11)

|  | ACR Classification criteria | Patients fulfilling (n) |
| --- | --- | --- |
| 1. Malar Rash  2. Discoid Rash  3. Photosensitivity  4. Mucosal Ulcers  5. Arthritis  6. Serositis  7. Renal Disorder  8. Neurologic Disorder  9. Hematologic Disorder  10. Immunologic Disorder  11. Antinuclear Antibody (ANA) | | 6 |
|  |  | 3 |
|  |  | 3 |
|  |  | 6 |
|  |  | 11 |
|  |  | 2 |
|  |  | 3 |
|  |  | 0 |
|  |  | 7 |
|  |  | 10 |
|  |  | 10 |

* Using the 1997 Revised ACR Classification Criteria for SLE.
