# Supplementary material for: Cerebral microvascular and microstructural integrity is regionally altered in patients with systemic lupus erythematosus
Source: Arthritis Res Ther. 2020 Jun 8;22:135. doi: 10.1186/s13075-020-02227-7 (PMC7281933; doi:10.1186/s13075-020-02227-7)
Supplement: Supplementary file 2 — Additional file 2: Table S2. Cognitive Assessment – Domains and Tests Used. Details of the various cognitive domains assessed for the study participants. [file 13075_2020_2227_MOESM2_ESM.docx]

SUPPLEMENTARY TABLE 2. Cognitive Assessment – Domains and Tests Used

| Cognitive Domain | Tests Used |
| --- | --- |
| 1. Working memory | - Wechsler Intelligence Scale for Children (WISC IV) Digit Span and Letter-Number Sequencing |
| 1. Psychomotor speed | - WISC IV Coding & Symbol Search - Conners' Continuous Performance Test (CPT) II reaction time parameter |
| 1. Attention | - Conners’ CPT II Stroop Color & Word Test |
| 1. Visuoconstructional ability | - Wechsler Abbreviated Scale of Intelligence Block Design Test - Kaufman Assessment Battery for Children - Second Edition Block Counting - Gestalt Closure |
